# Organ-Specific Differentiation of Human Adipose-Derived Stem Cells in Various Organs of Xenotransplanted Rats: A Pilot Study

**DOI:** 10.3390/life12081116

**Published:** 2022-07-25

**Authors:** Jung Ho Park, Yeon Ju Choi, So Young Kang, Hyunjeong Ju, Kyueng-Whan Min, Nan Young Kim, Ha Young Park, Eun Soo Kim, Mi Jung Kwon, Yong Joon Suh

**Affiliations:** 1 Department of Breast and Endocrine Surgery, Hallym University Sacred Heart Hospital, Anyang 14068, Korea; ringri@hallym.or.kr; 2 Research Cooperation Center, Hallym University, Anyang 14068, Korea; 41266@hallym.ac.kr; 3 Department of Pathology and Translational Genomics, Samsung Medical Center, Sungkyunkwan University School of Medicine, Irwon-dong, Gangnam-gu, Seoul 06351, Korea; sy500.kang@samsung.com; 4 Thermo Fisher Scientific Korea Ltd., 281, Gwangpyeong-ro, Gangnam-gu, Seoul 06349, Korea; heni7@naver.com; 5 Department of Pathology, Hanyang University Guri Hospital, Hanyang University College of Medicine, Guri 11923, Korea; kyueng@hanyang.ac.kr; 6 Hallym Institute of Translational Genomics and Bioinformatics, Hallym University Medical Center, Anyang 14068, Korea; honeyny@hallym.or.kr; 7 Department of Pathology, Busan Paik Hospital, Inje University College of Medicine, Busan 47392, Korea; mint@inje.ac.kr; 8 Department of Radiology, Hallym University Sacred Heart Hospital, Hallym University College of Medicine, 22, Gwanpyeong-ro 170 beon-gil, Dongan-gu, Anyang 14068, Korea; silwater@hallym.or.kr; 9 Department of Pathology, Hallym University Sacred Heart Hospital, Hallym University College of Medicine, 22, Gwanpyeong-ro 170 beon-gil, Dongan-gu, Anyang 14068, Korea

**Keywords:** adult stem cell, adipose-derived stem cell, direct intra-organ xenotransplantation, differentiation, rats

## Abstract

Adipose-derived stem cells (ADSCs) are potential therapeutics considering their self-renewal capacity and ability to differentiate into all somatic cell types in vitro. The ideal ADSC-based therapy is a direct injection into the relevant organs. The objective of this study was to investigate the viability and safety of intra-organ human ADSC (h-ADSC) xenotransplants in vivo. Subcutaneous adipose tissue from the abdominal area of 10 patients was sampled. h-ADSCs were isolated from adipose tissue samples and identified using immunofluorescence antibodies. Multi-differentiation potential assays for adipocytes, osteocytes, and chondrocytes were performed. Cultured h-ADSCs at passage 4 were transplanted into multiple organs of 17 rats, including the skin, subcutaneous layer, liver, kidney, pancreas, and spleen. The h-ADSC-injected organs excised after 100 days were examined, and the survival of h-ADSCs was measured by quantitative real-time polymerase chain reaction (qRT-PCR) using specific human and rat target genes. h-ADSCs confirmed by stem cell phenotyping were induced to differentiate into adipogenic, osteogenic, and chondrogenic lineages in vitro. All rats were healthy and exhibited no side effects during the study; the transplanted h-ADSCs did not cause inflammation and were indiscernible from the native organ cells. The presence of transplanted h-ADSCs was confirmed using qRT-PCR. However, the engrafted survival rates varied as follows: subcutaneous fat (70.6%), followed by the liver (52.9%), pancreas (50.0%), kidney (29.4%), skin (29.4%), and spleen (12.5%). h-ADSCs were successfully transplanted into a rat model, with different survival rates depending on the organ.

## 1. Introduction

Solid malignancies in breast and abdominal organs have been increasing worldwide [1]. For these malignancies, complete surgical resection with negative margins is still considered the central goal of treatment [2]. However, the attainment of clear surgical margins in patients with locally advanced tumors in the breast region and abdominal major organs may need extensive surgery and could cause uncovered tissue defects related to functional impairment as well as cosmetic issues; this is especially applicable in cases of large tumors or localization adjacent to critical anatomic structures [2]. In these circumstances, the application of adipose-derived stem cells (ADSCs) could be a potential alternative in regenerative medicine and reconstruction because of their ability for unlimited and self-renewal proliferation, immunomodulatory and proangiogenic features, and differentiation into progenitor cells or tissue-specific cells [3,4,5]. 

ADSCs have attracted attention as a preferable cell source owing to their ease of accessibility, clinically relevant abundance of adult mesenchymal stem cells, and reduced ethical issues compared to the use of embryonic stem cells [6,7]. Furthermore, ADSCs remain stable through extensive passages and can differentiate with low rates of apoptosis and strong proliferative capacity [8]. ADSCs have been applied in several therapeutic areas such as plastic, orthopedic, and cardiac surgery as well as in breast reconstruction [9]. Worryingly, possible oncologic risks associated with ADSC use have been proposed under extreme circumstances with the injection of co-cultured fatal cancer cell lines and h-ADSCs treated with ADSC conditioned medium in animal models [10,11], which is clearly different from the no-residual-tumor status obtained after complete surgical resection. Stem cell-based therapies for reconstruction after cancer would be safe if the patient is clinically disease-free [12]. Given that transplanted ADSCs in situ exert self-renewal capacity and the ability to differentiate into all somatic cell types [3], the optimal ADSC-based therapy would consist of a direct single-cell transplantation of ADSCs into the relevant organs [13,14] to generate organoids by mimicking human development or organ regeneration in vitro [15], which might ameliorate the possible safety issues. 

Several previous studies have exclusively targeted specific and limited organs. Nonetheless, there has been scarce information on simultaneously naive ADSC transplantation into multiple abdominal organs in vivo with long-term follow-up in the field of regenerative medicine of the subcutaneous fat, skin, liver, pancreas, kidney, and spleen, which are major organs in the field of abdominal and breast oncology surgery. Previous studies showed that intravenously injected human ADSCs (h-ADSCs) can migrate into injured atrial tissue and express a cardiomyocyte-like phenotype, indicating the viability of intravenous stem cell delivery [16]. Preliminary data revealed that direct intravenous infusion of autologous bone marrow-derived stem cells was feasible and safe during a short-term follow-up period [17]. In addition, the use of fetal calf serum or fetal bovine serum in cell culture may cause problems [18], particularly regarding the safety of xenogeneic components during heterologous stem cell transplantation. To date, most reports have relied on short-term observations (<4 weeks) after cell transplantation [16,17]. The long-term safety of the direct injection of h-ADSCs into various organs in rat models remains poorly understood. Further knowledge on in vivo direct stem cell injection in animal models could help optimize effective therapeutic strategies.

In this study, we evaluated the viability and potential safety of multiple intra-organ administrations of h-ADSCs in rat models.

## 2. Materials and Methods

### 2.1. Patient Population and Sample Collection

This study was performed in compliance with the tenets of the Declaration of Helsinki for experiments involving human tissues under the ethical approval issued by the Institutional Review Board (IRB) (NON2019-003). Samples of subcutaneous adipose tissue of the abdominal area were obtained from ten patients (mean age, 45 years; range, 27–71 years) undergoing excision of benign nodules after obtaining written informed consent in accordance with the IRB-approved protocol between August and September 2019. 

### 2.2. ADSC Isolation, Culture, and Identification

The ADSCs from adipose tissue were isolated as previously described [19]. Briefly, the fat tissues were minced with a sterile blade, washed with phosphate buffered saline (PBS) three times, and digested with 0.1% collagenase type 1 (Invitrogen, Carlsbad, CA, USA) in a shaking incubator at 250 rpm and 37 °C for 60 min. The digested tissues were then centrifuged at 1500 rpm for 10 min to remove the remaining adipose tissue and oil. The pellet was incubated with red cell lysis buffer (Roche) for 10 min and filtered using a cell strainer (100 μm, Falcon, Corning, NY, USA), followed by centrifugation at 2000 rpm for 5 min. The pellet was resuspended in PBS, filtered with a cell strainer (70 μm, Falcon), and centrifuged at 2000 rpm for 5 min. The supernatant was discarded, and the cell pellet was resuspended in low glucose Dulbecco’s modified Eagle medium (DMEM) medium containing 15% fetal bovine serum (FBS, Invitrogen, Thermo Fisher Scientific Inc., Waltham, MA, USA), 100 U/mL penicillin (Invitrogen), 100 μg/mL streptomycin (Invitrogen), and 2 mM L-glutamine (Invitrogen) and incubated at 37 °C and 5% CO_2_. After 48 h, the medium was changed and ADSC (passage 0) were maintained for 6–8 days. Passage numbers 2–5 of the ADSC were used for all experiments.

### 2.3. Phenotyping

For ADSCs phenotyping, samples were screened by immunofluorescence staining of cultured cells. The identification of ADSCs was carried out using antibodies against the specific cell surface antigens of ADSCs: positive [CD90 (sc-53456), CD29 (sc-9970, 1:500, Santa Cruz, CA, USA), and CD105 (sc-18893, endoglin; 1:500, Abcam)] and negative [CD31 (sc-18916, 1:300, Santa Cruz), CD45 (sc-1187; 1:50), and CD11b (14-0112-82, M1/70; 1:100, Abcam)] protein markers [20,21]. Cultured cells (passage 4) were seeded in a chamber slide (Lab-Tek, Thermo Fisher), fixed with 4% paraformaldehyde (PFA), and blocked in 3% bovine serum albumin (BSA; Sigma, St. Louis, MO, USA) in PBS (1X; 155 mM NaCl, 1 mM KH_2_PO_4_, 3 mM Na_2_HPO_4_-7H_2_O, pH 7.4; Sigma) containing 0.3% Triton X-100 (Sigma). Then, the cells were incubated with primary antibodies in 1% BSA in PBS containing 0.1% Tween-20 (Sigma) at 4 °C overnight. After washing, the cells were incubated with Alexa Fluor^®^ 555 goat anti-rabbit IgG (1:500, Invitrogen) for CD105 and Alexa Fluor ^®^ 555 donkey anti-rat IgG (1:1000, Invitrogen) for CD11b at room temperature for 1 h. Negative controls were treated with secondary antibodies only. Cell nuclei were counterstained with 4′,6′-diamidine-2-phenylindol (DAPI, Invitrogen, Carlsbad, CA, USA). Images of sections were obtained using a fluorescence microscope (Olympus, Tokyo, Japan).

### 2.4. Adipogenic, Osteogenic, and Chondrogenic Differentiation and Identification

To confirm the capacity of ADSCs to differentiate into adipogenic, osteogenic, and chondrogenic cells, ADSCs were differentiated in a specific differentiation medium.

To identify the adipogenic differentiation capacity of ADSCs, they were placed into 96-well plates at 3000 cells per well and cultured in DMEM or adipogenic differentiation medium (StemPro Adipogenesis Differentiation Kit, Thermo Fisher Scientific Inc.) for 14 days. The culture medium was changed every three days. On day 14 after induction for adipogenic differentiation, the cells were fixed with 4% PFA solution, washed with PBS, and stained with a solution of 0.5% (*w*/*v*) Oil Red O (Sigma) in 60% 2-propanol for 30 min to identify neutral lipid accumulation. The cells were rinsed three times with distilled water and observed under a microscope.

The osteogenic differentiation capability of the isolated cells was investigated using the protocol provided by the commercial differentiation kit (StemPro Osteogenesis Differentiation Kit, Thermo Fisher Scientific Inc.). ADSCs were cultured in a control growth medium (CGM) or osteogenic medium for 21 days and fixed with 4% PFA for 60 min. To detect mineralization, the cells were stained with 2% alizarin red (pH 4.1) at room temperature for 45 min, washed with PBS, and observed under a microscope. 

To examine the chondrogenic differentiation of ADSCs, a micromass culture of isolated ADSCs was performed. ADSCs (10^5^ cells/10 μL) were spotted in the center of the well of a 12-well culture plate and were incubated for 2 h without the addition of a medium. The isolated cells were incubated with CGM or a chondrogenic differentiation medium (low glucose DMEM), supplemented with 2 mM L-glutamine, 1× Insulin-Transferrin-selenim, 50 μM L-ascorbic acid, 40 μg/mL L-proline, 0.1 μM dexamethasone, and 10 ng/mL recombinant human transforming growth factor (TGF)-β3 for 28 days. After complete differentiation, the micromass cultures were fixed with 4% PFA, stained in the dark with 1% alcian blue solution for 1 h, and washed with PBS. 

### 2.5. Transplantation of Cells into Animal Organs

All animal study protocols and experimental procedures were reviewed and approved by the Institutional Animal Care and Use Committee (HMC 2019-1-0612-17) and carried out in accordance with the Guidelines for the Care and Use of Laboratory Animals published by the U.S. National Institute of Health. The animals used in this study included 20 young male Sprague Dawley rats (6–7 weeks old, weighing 198–253.5 g), which were purchased from the Jun Biotech Inc. (Daegu, Korea) and housed in micro-isolator cages at the Laboratory Animal Center in Hallym University Medical Center (Anyang, Korea). Throughout the study, all rats were kept in an environmentally controlled room at a constant temperature of 22 ± 1 °C with a relative humidity of 60%, with a 12 h light-dark cycle, and free access to food and water (Supplementary Figure S1). 

For all procedures (Supplementary Figure S2), the animals were anesthetized by the inhalation of a mixture of O_2_ and 2% isoflurane (Hana Pharm. Co., Seoul, Korea), and mechanically ventilated (136 bpm, tidal volume 0.15 mL). After anesthesia, the animals were skinned, cleaned, fixed using a rubber band on the rat’s extremities on the experimental table in a supine position, disinfected, and laid with sterile surgical towels. After skin preparation, the abdomen of the rats was opened with sterile manipulation. Peripheral organs such as the abdominal subcutaneous tissue layer, skin, liver, kidney, pancreas, and spleen were identified, and h-ADSCs were injected with clip marking. 

As the h-ADSC solution for injection was composed of 10^6^ cells per cc, the stem cell number for transplantation was 1 × 10^6^ cells per organ. Rats were randomly assigned to three groups (Supplementary Table S1): (I) a single organ injection group—four rats were transplanted with 1 mL h-ADSC solution to one organ (adipose tissue, liver, kidney, and skin); (II) five organ injection group—six rats were transplanted with 1 mL h-ADSC solution to five organs (adipose tissue, liver, kidney, skin, and pancreas); and (III) six organ injection group—seven rats were transplanted with 1 mL h-ADSC solution to five organs (adipose tissue, liver, kidney, skin, pancreas, and spleen). As a negative control group, three rats were injected with an equal volume of PBS alone into the subcutaneous tissue, liver, kidneys, pancreas, and spleen. 

The animals were monitored postoperatively for immediate reactions (e.g., allergic reactions [tachycardia, fever, skin eruption], local complications [hematoma or local infection at the injection site], vascular obstruction [tachypnea, oliguria, or peripheral vascular insufficiency], systemic complications [systemic infections]), and long-term adverse events (e.g., tumor formation and zoonoses including myoclonus, rapidly progressing dementia, or ataxia), or abnormal behavior. 

After 100 days, all rats were euthanized using a CO_2_ chamber. The h-ADSC-injected organs were excised after 100 days and reviewed by pathologists for the identification and analysis of stem cell implants. 

The primary outcome measure was the long-term safety profile over 3 months. We monitored mortality of any cause and serious adverse events possibly related to xenotransplantation of h-ADSCs into rats. The secondary outcome measure was the settlement of h-ADSCs with organ-specific differentiation. 

### 2.6. Detection of Human and Rat Cells by Quantitative Real-Time Polymerase Chain Reaction

Quantitative real-time polymerase chain reaction (qRT-PCR) can be used to identify the presence of specific human and rat materials through a biochemical process of amplification using enzymes and based on specific target recognition. Therefore, the presence of h-ADSCs in rat organs was measured by qRT-PCR using TaqMan Gene Assays (Thermo Fisher Scientific, USA). The following rat- and human-specific gene primer pairs were used as the reference assay: β-actin (ACTB) (Rn_07315855_s1; Thermo Fisher Scientific) and RNaseP (Thermo Fisher Scientific; Part No. 4403326), respectively. For each human-specific primer pair validation, we performed a no template control (NTC) and normal genomic DNA (Roche, Basel, Switzerland; Part No. 11 691 112 001). PCR amplifications were performed in triplicate using the following conditions on QuantStudio 3 (Thermo Fisher Scientific, USA): 2 min at 50 °C and 10 min at 94 °C, followed by 40 cycles at 95 °C for 15 s and 60 °C for 60 s. Quantitative values were obtained from the cycle number (Ct value) (Thermo Fisher Scientific), according to the manufacturer’s instructions. 

### 2.7. Histological Evaluation for Transplanted Tissues

For light microscopic examination, the h-ADSC-injected organs resected from experimental animals were fixed in 10% buffered formalin, embedded in paraffin, and cut into 1-µm sections (this thickness was the minimum allowed by the microtome in the laboratory). The sections were stained with hematoxylin and eosin (H&E). All the sections were examined under a light microscope and photographed. The purpose of the H&E-stained microscopic examination was to observe any morphological changes (including immediate and long-term adverse microscopic reactions) in the tissues of the rats transplanted with h-ADSCs. 

### 2.8. Statistical Analysis

Results and demographic data are presented as mean ± standard deviation (SD) deviation or as numbers (n) and percentages. The SPSS statistical software (ver. 20) (IBM Corp., Armonk, NY, USA) was used for all the statistical analyses. *p* < 0.05 was considered statistically significant.

## 3. Results

### 3.1. Identification of ADSC by Immunofluorescence

**Figure 1:**
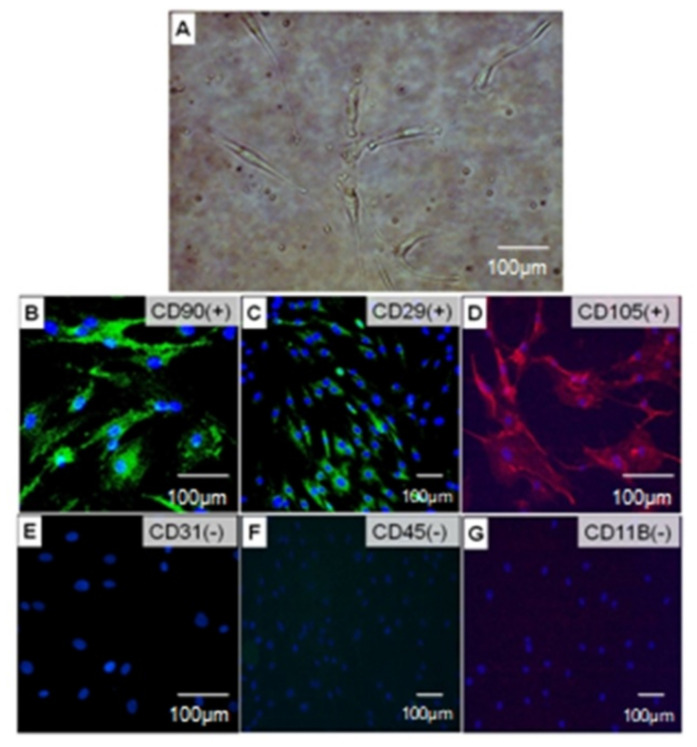
(**A**) Representative micrographs of human adult adipose tissue-derived stem cell (h-ADSC) morphology in day 4 under light microscopy, magnification ×400. Immunofluorescence staining of cultured cells demonstrates the features of h-ADSCs positive for CD90 (**B**), CD29 (**C**), and CD105 (**D**) and negative for CD31 (**E**), CD45 (**F**), and CD11b (**G**).

In the present study, h-ADSCs cultured in a medium containing 20% fetal bovine serum showed a characteristic spindle-shaped and fibroblast-like morphology (Figure 1A).

The isolated h-ADSCs were identified based on the expression of h-ADSC surface antigens (CD90, CD29, and CD105) and negative markers (CD31, CD45, and CD11b) assessed by immunofluorescence staining. Compared with the control, we found that the analyzed cells were positive for stem cell markers (CD90, CD29, and CD105) (Figure 1B–D) but negative for endothelial cells or hematopoietic lineage markers (CD31, CD45, and CD11b) (Figure 1E–G). Control cells were negative for CD90, CD29, CD105, CD31, CD45, and CD11b. These results revealed that cultured h-ADSCs were homogeneous and did not contain endothelial cells or hematopoietic lineages, which is consistent with previous reports [20,21].

**Figure 2:**
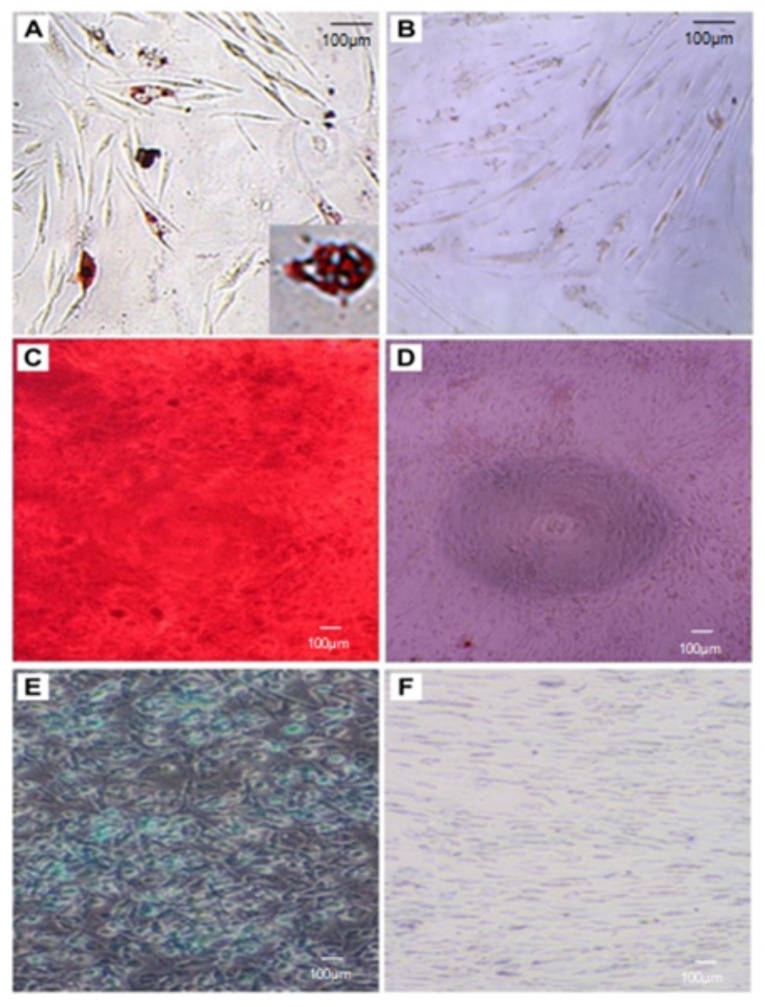
Cultured cells were differentiated into adipocytes identified by Oil Red O staining (**A**), osteocytes by alizarin red S staining (**C**), and chondrocytes by alcian blue staining (**E**). The respective control cells (**B**,**D**,**F**) in the DMEM medium were negative.

To examine the differentiation capability of h-ADSCs, cells cultured at passage 4 were induced to differentiate into adipocytes, osteocytes, and chondrocytes (Figure 2). Adipogenesis was confirmed by lipid droplet formation in the cytoplasm stained red with the Oil Red O dye. Osteogenesis was also identified by the presence of black calcium deposits in cultures stained red by alizarin red staining. Chondrogenesis was detected by alcian blue staining of proteoglycans synthesized by chondrocytes differentiated from h-ADSCs. These results showed that the analyzed cells were h-ADSCs with multiple differentiation capabilities.

### 3.2. Transplantation of h-ADSCs into Rats

**Figure 3:**
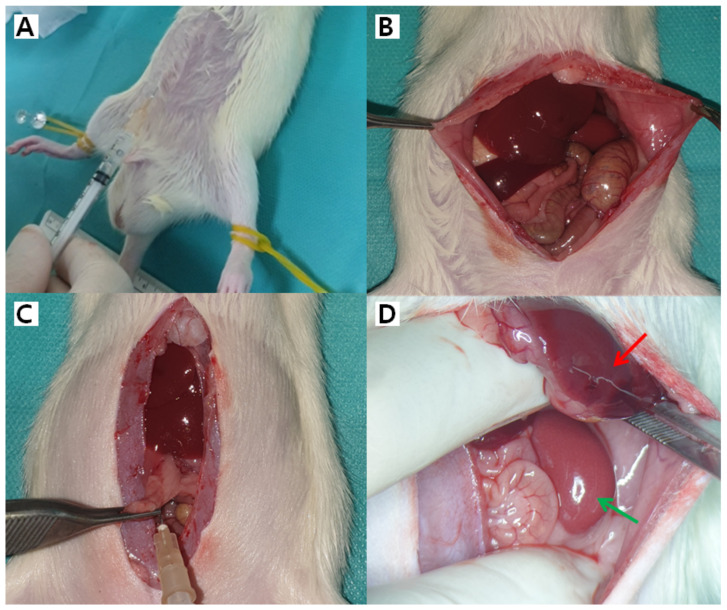
Xenotransplantation of human adult adipose tissue-derived stem cells (h-ADSCs) in a rat model. (**A**) h-ADSCs were injected in a sterile manner into the abdominal skin and subcutaneous tissue. (**B**) The intraperitoneal cavity was opened, and abdominal organs were exposed. (**C**,**D**) h-ADSC was injected into the pancreas (**C**), liver (red arrow), and kidney (green arrow).

**Figure 4:**
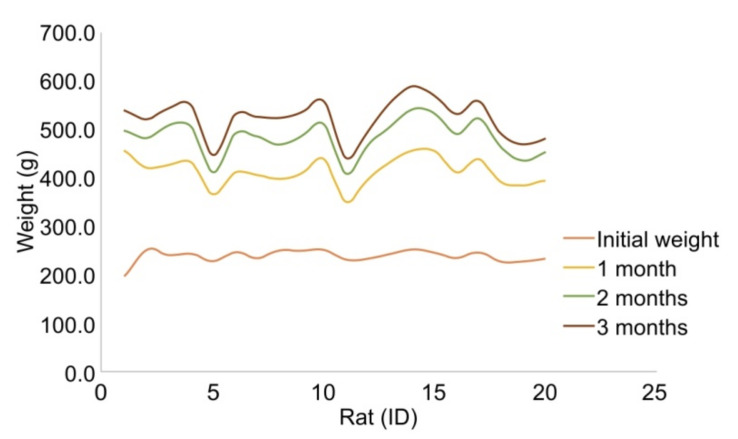
All 20 rats enrolled in the study were healthy during the 100 days of the study. The rats exhibited normal weight gain and behavior.

**Figure 5:**
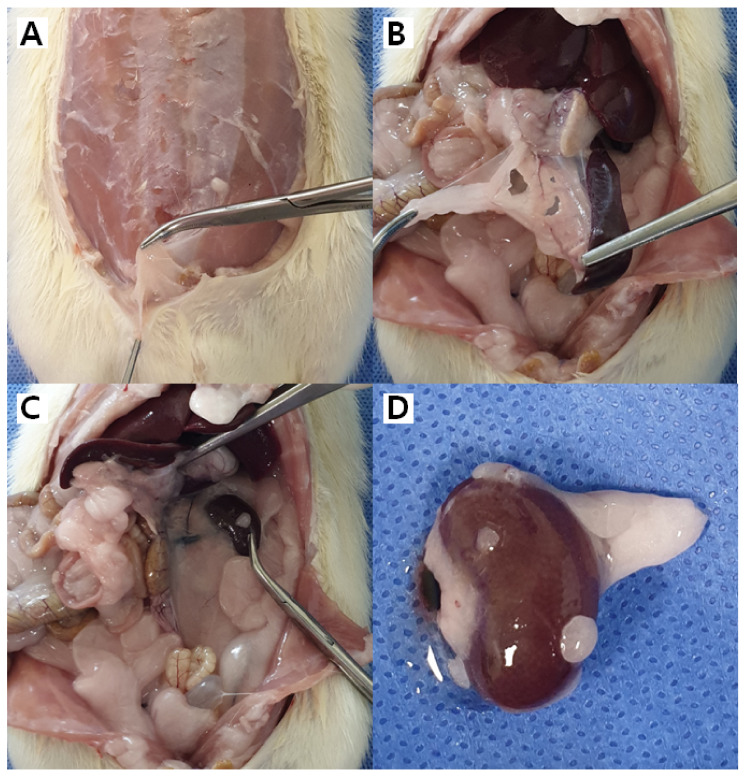
Gross findings of the injected human adult adipose tissue-derived stem cells (h-ADSCs) in a rat model. After euthanasia, injected h-ADSCs were harvested from abdominal subcutaneous tissue (**A**), and abdominal organs (**B**,**C**). Granulation tissue was identified on the renal injection site (**D**).

We next investigated the fate of h-ADSCs in vivo, depending on the organ. h-ADSCs at passage 4 were injected into the subcutaneous fat, skin, liver, kidney, pancreas, and spleen of 17 rats, while normal saline was injected into the corresponding organs of three control rats (Figure 3). All 20 animals enrolled in the study were healthy during the study period, exhibited normal weight gain, and displayed normal behavior without any signs of immediate reaction or long-term adverse events throughout the study (Figure 4). During follow-up, mortality from any cause and serious adverse effects, possibly related to xenotransplantation of h-ADSCs into rats, were not observed. The h-ADSC-injected skin, subcutaneous fat, liver, kidney, pancreas, and spleen were examined in H&E-stained sections 100 days after either cell transplantation or saline injection. All organs were grossly normal except for the granulation tissue identified at the renal injection site. There were no obvious or microscopic morphological differences between the control and experimental rat groups (Figure 5). The normal structure of each organ was microscopically observed in both groups; no neoplastic changes, inflammatory cell aggregates, or immuno-rejection were observed in the h-ADSC-injected areas.

### 3.3. Human- and Rat-Specific Reference Genes in h-ADSC-Injected Organs

**Figure 6:**
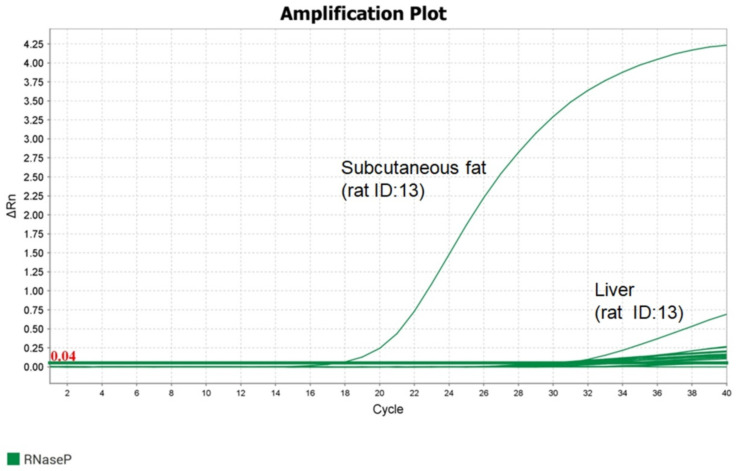
Quantitative real-time-polymerase chain reaction (qRT-PCR) using specific human and rat target genes for the human adult adipose tissue-derived stem cells (h-ADSC)-injected rats’ organs. The RNaseP gene was amplified in the subcutaneous tissue and liver of the rat (ID: 13) injected with h-ADSCs after 100 days.

We used qRT-PCR to evaluate the presence of ACTB in rats and the human-specific gene (RNaseP) primer pairs to detect the presence of h-ADSCs in the injected rat organs (Figure 6). The detection of RNaseP in the h-ADSC-injected rat organs after 100 days is summarized in Table 1. The most common organ for human-specific gene detection was the subcutaneous fat (70.6%), followed by the liver (52.9%), pancreas (50.0%), kidney (29.4%), skin (29.4%), and spleen (12.5%). These in vivo data were consistent with the results obtained using h-ADSCs.

## 4. Discussion

In the current study, we successfully isolated h-ADSCs from lipoaspirate tissue samples, which were confirmed by immunofluorescence and assessment of adipogenic, chondrogenic, and osteogenic differentiation potential. These h-ADSCs showed specific cell surface stem cell markers (CD90, CD29, and CD105) but negative endothelial cell or hematopoietic lineage markers (CD31, CD45, and CD11b), which is consistent with the expression profiles of ADSCs [7,20,21]. The highlights of our study include the direct simultaneous in vivo injection of naive h-ADSCs into up to 6 abdominal organs and related tissues that are the most frequently encountered in abdominal surgery as well as the related reconstruction, which may further the understanding of h-ADSCs engraftment that was confined to several major organs in previous studies [9]. 

ADSC implantation in the organ is a complex process involving organ-specific cell differentiation, proliferation, and cell survival, which are essential for successful transplantation [22]. The direct incorporation of stem cells into organs may enhance organ-specific differentiation [23]. We observed the presence of h-ADSCs in all six injected organs (skin, subcutaneous fat, liver, pancreas, kidney, and spleen) injected with a single cell suspension containing 1 × 10^6^ cells per cc, which was confirmed through the presence of human-specific genes using qRT-PCR. This suggests that transplanted h-ADSCs were engrafted into various rat organs. The h-ADSCs did not disappear and were able to survive 100 days after transplantation. Similarly, in the heart, the direct transplantation of ADSCs into the target organ has been demonstrated to lead to enhanced cell retention and improved organ function compared to intravascular infusion [24]. In many cases, the survival of transplanted stem cells is too low to explain the significant organ improvement. The transplanted stem cells have been shown to release soluble factors including cytokines and growth factors in a paracrine fashion, mediating tissue repair, remodeling, and metabolism as well as endogenous regeneration via the activation of resident organ stem cells [25].

The engrafted survival rate varied in this study. Subcutaneous fat was the highest site of human RNase P detection. The human gene was detected in 50% of liver or pancreas samples and 30% of kidney or skin samples. The spleen had the lowest detection rate. These results indicate that the successful settlement proportions of the injected h-ADSCs vary depending on the recipient rat organ. Since h-ADSCs were originally obtained from adipose tissue sources, the settlement rate of h-ADSCs may be highest in the same cell-derived origin organ sites. The leading causes of the poor survival of stem cells in vivo are connected to anoikis, potential immune rejection, and oxidative damage- mediating apoptosis [26]. To boost the stem cell survival and therapeutic function after transplantation, pro-survival cocktail injections, microenvironmental preconditioning including hypoxia, heat shock, and exposure to oxidative stress, aggregate formation, and hydrogel encapsulation have been modulated to lessen cell apoptosis in vivo while sustaining cellular biological functions [6,25,27]. However, stem cell manipulation may possibly be carcinogenic, potentially increasing the risk of additional harm in cancer patients [9,28].

Previous studies have demonstrated that transplantation of ADSCs into the mouse liver transdifferentiated into hepatic lineage cells [14,29], suggesting that ADSCs are a potential source of undifferentiated cells for liver cell transplantation. However, there has been scarce research regarding other organs [16,22,30]. When transplanted in vivo into specific organs, ADSCs demonstrate a high regenerative potential and impart to specific cell formation and to the repletion of the blood vessels probably due to their intrinsic multipotency [31,32]. Mesenchymal cells of human adult tissue can be directed toward an organ-specific cell phenotype when in contact with differentiating primary organ progenitor cells in vitro and in vivo [32,33,34], whereas ADSCs are likely unable to produce new muscle fibers de novo or even to promote a complete skeletal muscle program [35,36]. In this context, ADSC-based therapy may contribute to the further development of tissue engineering to recover tissue defects or damage after oncologic surgery leading to cosmetic problems [37].

The safety and reliability of heterologous h-ADSCs transplantation into animals or vice versa remain controversial [8,38]. Due to the potentially different in vivo microenvironment in rats compared with humans, it may be hard to assess the interaction between ADSC-based therapy and the immune system [39]. We observed that h-ADSCs collected from 10 different donors did not cause any immediate or delayed fatal reactions or death when those stem cells were injected into 17 rats over 3 months. All rats displayed normal health and behavior. These results indicate that h-ADSC-transplanted rat organs may be stable and safe from inflammatory or immunogenic reactions [13]. Mesenchymal stem cells may have anti-inflammatory and immunomodulatory properties [40], allowing immunosuppressive drug minimization, and inducing immune tolerance towards the transplanted organ [8,41]. Intra-organ administration of 1 × 10^6^ xenotransplanted h-ADSCs into six rat organs did not elicit any detectable inflammatory or necrotizing reactions within the organs. We also found no gross or microscopic adverse morphological changes. Xenotransplantation of h-ADSCs did not adversely affect the anatomical structures in naïve rat organs. These results suggest the potential safety of multiple intra-organ administrations of h-ADSCs in xenotransplanted rat models. Our results support recent biological tissue engineering approaches by stem cell transplantation, which automatically regulate and achieve individually specified organ phenotypes and functions that adapt to the patient’s physiological requirements [30]. The trans-lineage differentiation power and peculiar immunogenic features of ADSCs have identified them as a potential substitute for cellular transplantation in regenerative medicine [42]. However, caution must be exercised regarding the malignant potential of ADSC injections as previous studies have shown that ADSCs may enhance tumor initiation and growth in breast and colon cancer cells, stimulating cancer cells to secrete interleukin-6 in a paracrine manner to enhance their malignant properties [9].

Our study has limitations. h-ADSCs were successfully transplanted into this rat model and the overall behavioral and morphological parameters were normal. The potential clinical significance of this study may include the relatively high survival rates of directly injected ADSCs into multiple clinically relevant organs mediated by xenotransplanted rat models, which allowed us to recognize their long-term survivals using the human- and rat-specific target genes. Nonetheless, we could not investigate the differentiation control of h-ADSCs in vivo and the functional ability of the engrafted h-ADSCs, which requires further research. 

In summary, human ADSCs injected into the normal organs of rats survived and engrafted at varying rates depending on the recipient rat organ after 100 days. The current study highlights the practical application of xenotransplantation of h-ADSCs directly into various organs of rats. 

## Figures and Tables

**Table 1:** Detection rate of human-specific gene (RNaseP) in rats’ organs after 100 days.

	RNaseP Positive	RNaseP Negative	Fail
Subcutaneous fat (n = 17)	12 (70.6%)	4 (23.5%)	1 (5.9%)
Skin (n = 17)	5 (29.4%)	10 (58.8%)	2 (11.8%)
Liver (n = 17)	9 (52.9%)	8 (47.1%)	0 (0%)
Kidney (n = 17)	5 (29.4%)	12 (70.6%)	0 (0%)
Pancreas (n = 4)	2 (50.0%)	2 (50.0%)	0 (0%)
Spleen (n = 16)	2 (12.5%)	14 (87.5%)	0 (0%)

## Data Availability

The data used to support the findings of this study are available from the corresponding author upon request.

## Supplementary Materials

The following supporting information can be downloaded at: https://www.mdpi.com/article/10.3390/life12081116/s1, Figure S1: Rats had ad libitum access to water and food and were housed under adequate temperature (23 °C) and a 12-h light-dark cycle. Figure S2: (A) During direct intra-organ xenotransplantation of human adipose-derived stem cells (h-ADSCs), rats were anesthetized and mechanically ventilated. The rat was fixed in a supine position using a rubber band on the extremities on the experimental table, disinfected, and laid with sterile surgical towels. (B) After skin preparation, rat abdomens were opened using a sterile procedure. Table S1: Summary of weight changes and study profile of 20 study and control rats throughout the study period.
