# Supplementary material for: Organ-Specific Differentiation of Human Adipose-Derived Stem Cells in Various Organs of Xenotransplanted Rats: A Pilot Study
Source: Life (Basel). 2022 Jul 25;12(8):1116. doi: 10.3390/life12081116 (PMC9330795; doi:10.3390/life12081116)
Supplement: Supplementary file 1 [file life-12-01116-s001.zip › life-1796819-supplementary.pdf]

Supplemental Materials

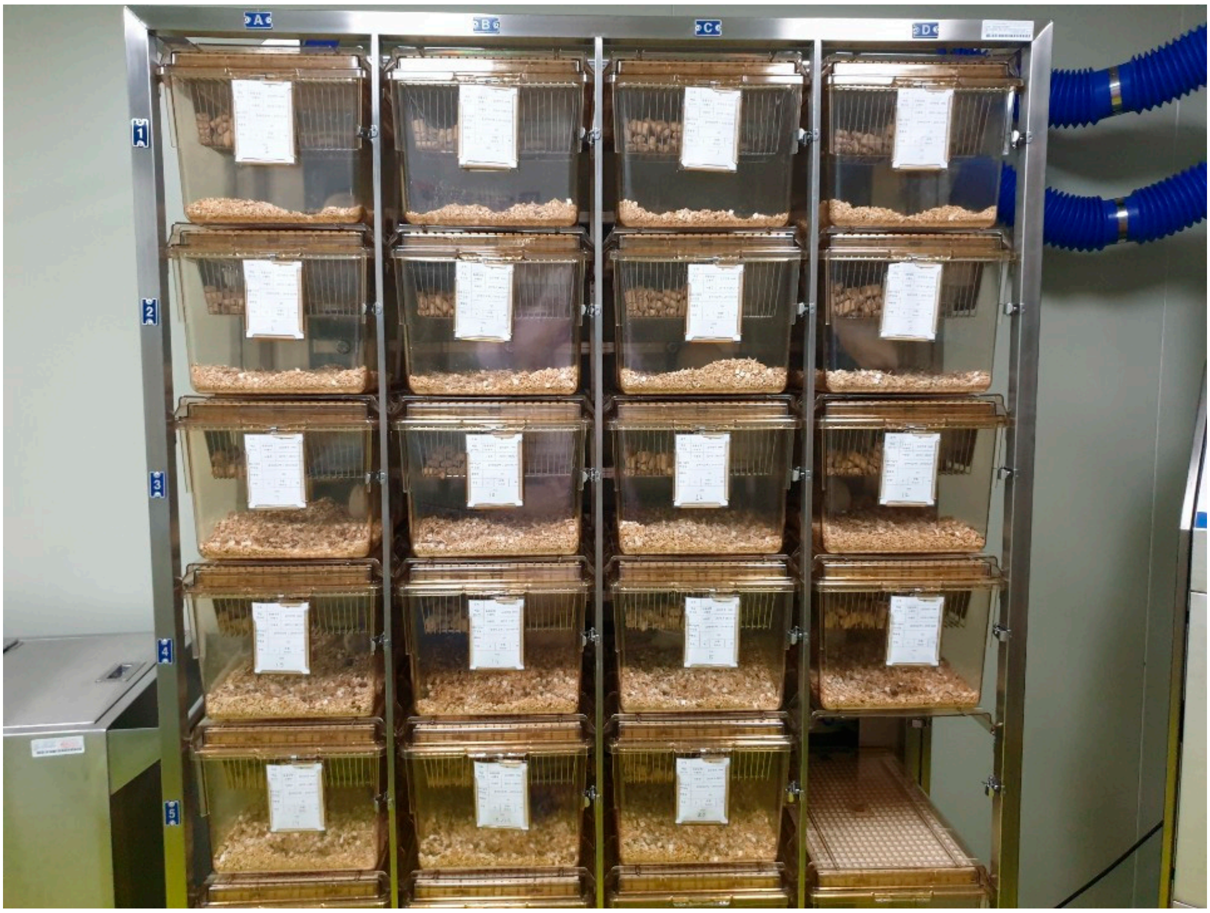

Figure S1. The rats had ad libitum access to water and food and were housed under adequate temperature (23℃) and a 12-hour light-dark cycle.

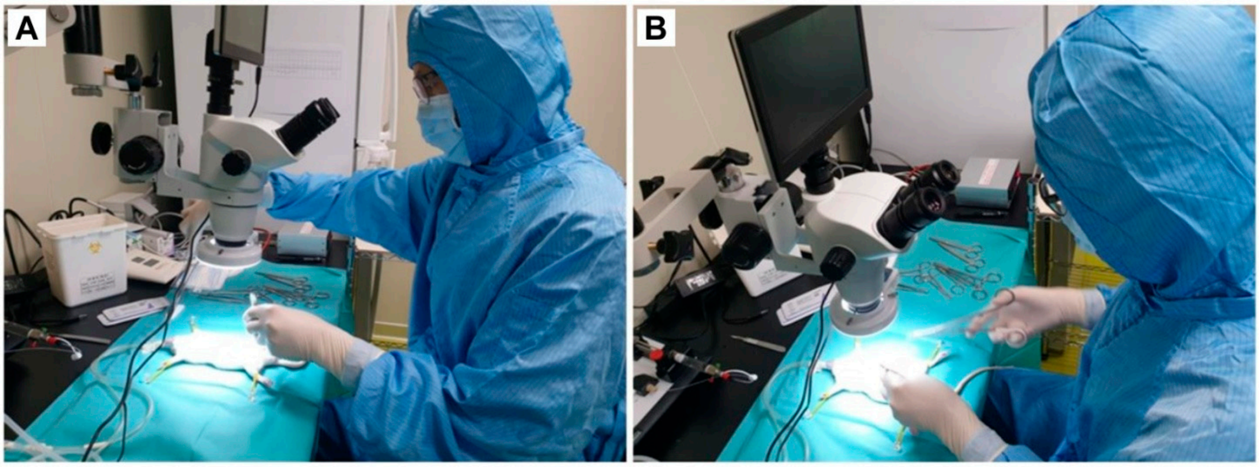

Figure S2. (A) During direct intra-organ xenotransplantation of human adipose-derived stem cells (h-ADSCs), rats were anesthetized and mechanically ventilated. The rat was fixed using a rubber band on the extremities on the experimental table in supine position, disinfected, and laid with sterile surgical towels. (B) After skin preparation, the rat abdomens were opened using sterile procedure.

Table S1. Summary of weight changes and study profile of 20 study- and control- rats throughout the study period.

ID	1	2	3	4	5	6	7	8	9	10	11	12	13	14	15	16	17	18	19	20
Age (weeks)	6 + 2	7 +1	7 +1	7 +1	7 +1	7 +1	7 +1	7 +1	7 +1	7 +1	7 +1	7 +1	7 +1	7 +1	7 +1	7 +1	7 +1	7 +1	7 +1	7 +1
Injection	10^6	10^6	10^6	10^6	10^6	10^6	10^6	10^6	10^6	10^6	10^6	10^6	10^6	10^6	10^6	10^6	10^6	- (control)	- (control)	- (control)
FAT	Y				Y	Y	Y	Y	Y	Y	Y	Y	Y	Y	Y	Y	Y	N	N	N
LIVER		Y			Y	Y	Y	Y	Y	Y	Y	Y	Y	Y	Y	Y	Y	N	N	N
KIDNEY			Y		Y	Y	Y	Y	Y	Y	Y	Y	Y	Y	Y	Y	Y	N	N	N
SKIN				Y	Y	Y	Y	Y	Y	Y	Y	Y	Y	Y	Y	Y	Y	N	N	N
PANCREAS					Y	Y	Y	Y	Y	Y	Y	Y	Y	Y	Y	Y	Y	N	N	N
SPLEEN											Y	Y	Y	Y	Y	Y	Y	N	N	N
Control	N	N	N	N	N	N	N	N	N	N	N	N	N	N	N	N	N	Y	Y	Y
Marking	Y	Y	Y	Y	Y	Y	Y	Y	Y	Y	Y	Y	Y	Y	Y	Y	Y	Y	Y	Y
Weight (g)	198.0	252.0	241.5	244.0	229.5	247.0	236.0	251.0	250.5	252.5	232.5	234.5	244.0	253.5	247.0	236.0	246.5	229.0	228.0	234.0
1 month	456.5	422.0	427.5	432.5	367.0	411.0	406.0	398.0	409.0	439.0	351.0	399.0	432.5	456.5	456.5	412.0	439.0	392.0	385.5	395.0
2 month	499.5	484.0	510.0	507.0	413.0	492.5	487.0	470.0	487.5	512.5	410.5	470.5	507.0	543.5	536.0	492.0	524.5	465.5	437.0	454.0
3 month	540.5	523.0	546.0	549.5	449.0	532.0	527.0	524.5	536.5	559.0	443.5	499.0	557.0	589.0	571.0	533.0	559.0	493.0	471.0	482.0
